# Galvanic Replacement Reaction as a Route to Prepare Nanoporous Aluminum for UV Plasmonics

**DOI:** 10.3390/nano10010102

**Published:** 2020-01-04

**Authors:** Denis Garoli, Andrea Schirato, Giorgia Giovannini, Sandro Cattarin, Paolo Ponzellini, Eugenio Calandrini, Remo Proietti Zaccaria, Francesco D’Amico, Maria Pachetti, Wei Yang, Hai-Jun Jin, Roman Krahne, Alessandro Alabastri

**Affiliations:** 1 Istituto Italiano di Tecnologia, via Morego 30, I-16163 Genova, Italypaolo.ponzellini@iit.it (P.P.); eugenio.calandrini@iit.it (E.C.); remo.proietti@iit.it (R.P.Z.); roman.krahne@iit.it (R.K.); 2 Deparment of Physics, Politecnico di Milano, Piazza L. da Vinci 32, I-20133 Milan, Italy; 3 EMPA Federal Swiss research Institute, 9014 St. Gallen, Switzerland; giorgia.giovannini@empa.ch; 4 ICMATE—CNR, Corso Stati Uniti 4, 35127 Padova, Italy; sandro.cattarin@cnr.it; 5 Cixi Institute of Biomedical Engineering, Ningbo Institute of Industrial Technology, Chinese Academy of Sciences, 1219 Zhongguan West Road, Ningbo 315201, China; 6 Elettra Sincrotrone Trieste S.C.p.A., S.S. 14 km 163,5 in Area Science Park, 34149 Basovizza TS, Italy; francesco.damico@elettra.eu (F.D.); maria.pachetti@elettra.eu (M.P.); 7 Department of Physics, University of Trieste, Via Alfonso Valerio 2, 34127 Trieste, Italy; 8 Shenyang National Laboraory for Materials Science, Institute of Metal Research, Chinese Academy of Sciences, 72 Wenhua Road, Shenyang 110016, China; wyang15s@imr.ac.cn (W.Y.); hjjin@imr.ac.cn (H.-J.J.); 9 Department of Electrical and Computer Engineering, Rice University, 6100 Main Street MS-378, Houston, TX 77005, USA; alessandro.alabastri@rice.edu

**Keywords:** plasmonic, ultraviolet, nanoporous, aluminum, SERS, metal enhanced fluorescence

## Abstract

There is a growing interest in extending plasmonics applications into the ultraviolet region of the electromagnetic spectrum. Noble metals are commonly used in plasmonic, but their intrinsic optical properties limit their use above 350 nm. Aluminum is probably the most suitable material for UV plasmonics, and in this work we fabricated substrates of nanoporous aluminum starting from an alloy of Al_2_Mg_3_. The porous metal is obtained by means of a galvanic replacement reaction. Such nanoporous metal can be exploited to achieve a plasmonic material suitable for enhanced UV Raman spectroscopy and fluorescence. Thanks to the large surface to volume ratio, this material represents a powerful platform for promoting interaction between plasmonic substrates and molecules in the UV.

## 1. Introduction

Collective oscillations of electron waves at the metal/dielectric interface, also known as Surface Plasmons, find extensive applications in different fields such as surface-enhanced Raman spectroscopy (SERS), metal-enhanced fluorescence (MEF), photocatalysis, etc. [1]. Plasmonics and its application have been mainly focused on visible and infrared spectral regions because of the optical properties of noble metals (Au and Ag) that are typically used. In fact, interband transitions introduce a dissipative channel for Au and Ag plasmon resonances at wavelengths shorter than 550 and 350 nm, respectively. On the contrary, during the last decade there has been an increasing interest in extending plasmonic effects down to UV and deep-UV (DUV) wavelengths [2,3,4,5]. UV and DUV excitations can be utilized to perform Raman spectroscopy on biomolecules that have small Raman cross sections in the visible and NIR regions [6,7,8,9]. Additionally, important biomolecules (such as some amino-acids) have intrinsic fluorescence in the UV region [10], and a platform able to enhance the detection limit thanks to plasmonic effects is highly appealing in biosensing [11,12,13]. Several alternative materials have been investigated during the recent years; among them magnesium [14], gallium [15,16,17,18,19], indium, rhodium [20,21,22,23,24], and aluminum (Al) [4,22,25,26,27]. Al in particular is still extremely interesting also thanks to its low cost and large abundance. It has been the object of extensive researches and has demonstrated interesting performances in the UV and DUV regions [28]. In order to use Al to generate localized surface plasmonic resonances (LSPR) in the UV spectral region, small metallic features/nanostructures must be prepared. Such structures can be designed with the help of electron beam lithography (EBL) and focused ion beam (FIB) lithography or achieved with different methods of synthesis of nanoparticles [29,30]. An alternative approach to generate LSPR is based on porous metal films that, during the last decade, have attracted increasing interest due to their unique, very high specific surface area. Chemical dealloying is the typical procedure to prepare nanoporous films [31]. Unfortunately, chemical synthesis of nanoporous Al in aqueous electrolytes is challenging because Al is very reactive and nanoscale Al ligaments may be rapidly fully oxidized. Very recently, H-J. Jin et al. [32], and J. S. Corsi et al. [33] demonstrated that it is possible to prepare porous Al structures by means of galvanic replacement reaction (GRR) and electrochemical dealloying, respectively, starting from an alloy of Al_2_Mg_3_. In particular, GRR involves a dissolution of sacrificial metals via galvanic oxidation and concurrent precipitation of second metals onto the sacrificial one via galvanic reduction of metallic precursor cations [34]. In a recent work, we reported on the preparation of Mg/Al nanoporous film under mild oxidation conditions. The films were obtained by submitting a Mg_x_Al_1-x_ alloy to a selective dissolution in acetic acid in methanol, that allows partial retention of metallic Al-Mg [7]. Unfortunately, the high reactivity to oxidation of Al and Mg does not allow to prepare porous metals with oxygen contents below 14%. Moreover, it has not been possible to achieve porous metals in pure Al phase.

Here we report on the synthesis of bulk nanoporous Al (NPA) as a platform for UV plasmonics. We will show how the porous matrix can support LSPR enabling significant enhancement in both fluorescence and Raman spectroscopy. Finally, numerical simulations will be used to describe the effect.

## 2. Materials and Methods

*Materials*: 7-hydroxil-4-coumarin acetic acid 97%, Aluminum Chloride (AlCl_3_) anhydrous, powder, 99.99% trace metals basis, 1-Ethyl-3-methylimidazolium chloride 98% [EMIM]Cl, *N*-(3-Dimethylaminopropyl)-*N*′-ethylcarbodiimide hydrochloride BioXtra (EDC hydrochloride), *N*-Hydroxysuccinimide 98% (NHS), (3-Aminopropyl)triethoxysilane 99% (APTES), Dimethyl sulfoxide anhydrous, ≥99.9% (DMSO). All mentioned chemicals were purchased from Sigma-Aldrich (St. Louis, MO, USA). Acetone, anhydrous (max. 0.01% H_2_O) ≥99.8% was purchased from VWR chemicals (Radnor, Pennsylvania, USA).

*Galvanic Replacement Reaction (GRR)*: The preparation of the NPA film started from an Al_2_Mg_3_ alloy prepared by melting high purity Al (>99.9%) and Mg (>99.9%) in a resistance furnace under Ar atmosphere. The NPA was prepared by means of GRR by immersing the Al_2_Mg_3_ sample in an [EMIM]+Al_2_Cl_7_-ionic liquid, which was prepared by mixing AlCl_3_ and 1-ethyl-3-methylimidazolium chloride, [EMIM]Cl at room temperature with a molar ratio of 2:1. The sample has been kept immersed in the ionic liquid for 6 days in order to ensure the formation of the bulk porous structure.

*Etching by chemical dealloying (acid treatment)*: Three additional samples of Al_2_Mg_3_ alloy prepared by melting were dipped in a 1 M methanol solution of acetic acid for 30 min; 3 and 7 h, respectively. The samples were then washed with methanol. Immediately after the acidic treatment, the samples were brought inside the glovebox.

*Preparation of standard reference samples*: Three reference samples have been used: Al_2_O_3_, smooth Al film, and rough Al thin film. For Al_2_O_3,_ we used a 500 µm thick commercial ceramic substrate (Semiconductor wafer Inc., Hsinchu, Taiwan). A smooth Al film has been deposited by means of electron beam evaporation on Silicon with a final thickness of 100 nm. Rough Al samples following a procedure described in [7,35].

*X-ray diffraction (XRD)* were performed by means of a PANalytical Empyrean X-ray diffractometer equipped with a 1.8 kW CuKα ceramic X-ray tube, PIXcel3D 2 × 2 area detector and operating at 45 kV and 40 mA. The diffraction patterns were collected in air at room temperature using Parallel-Beam (PB) geometry and symmetric reflection mode. XRD data analysis was carried out using HighScore 4.18 software from PANalytical (Malvern, United Kingdom).

*Energy-dispersive spectroscopy (EDS)* was performed within a JEOL JSM-7500LA SEM (JEOL, Tokyo, Japan), equipped with a cold field-emission gun (FEG), operating at 5 kV acceleration voltage. We measured the film composition through an Oxford instrument EDS setup (X-Max, 80 mm^2^). The measurements were performed at 8 mm working distance, 5 kV acceleration voltage, and 15 sweep count for each sample. EDS spectra from three different positions have been collected for each sample. In order to analyze the spectra, we used Aztec 1.2 software^®^ (Abingdon, UK), with automatic calibration of the standards and background subtraction. The instrument was calibrated with a Microanalysis Standard As-02756-AB 59 Metals & Minerals Carousel Serial HM, by SPI. For all the elements, we analyzed the K-alpha lines. For each element, the composition percentages measured in different positions differed one from the other by less than 1%.

*Samples functionalization with APTES:* The samples were shaken overnight, at room temperature, in 4% solution of APTES in acetone inside glove box. They were then washed in acetone and dried inside the glove box.

*Dye activation and attachment on the substrate*: EDC (0.02 mmol) was added to a solution of 7-hydroxyl-4-coumarin acetic acid (0.01 mmol) in DMSO (1 mL), reaching 10 mM as final concentration. The reaction was stirred at room temperature for 15 min. The solution was stirred for additional 45 min after addition of NHS (0.03 mmol). After 1 h, the mixture was diluted in acetone, reaching a final concentration of 1 mM of dye. 1 mL of this solution was added to the substrates in a 3 mL glass vial. The vials were shaken at room temperature in glove box overnight. The substrates were carefully withdrawn and washed with acetone. The remaining solution of coumarin was kept for dye quantification: The absorbance at 360 nm was used to determine the amount of coumarin present in each solution using as reference 1 mM solution of activated coumarin. The obtained data were used to estimate the amount of coumarin covalently bound to the substrate surface. Dye quantification was preferentially achieved by measuring dye absorption since this value is not affected by self-quenching, life-time, and fluorescence decay which characterize instead fluorescence measurements.

*Fluorescence measurement*: Once washed, all substrates were placed in a plate, a drop of phosphate buffer solution (PBS) was added on the surface in order to make a measurement under wet conditions, and the fluorescence spectrum was recorded with a 360 nm excitation wavelength (λ_ex_). A Tecan Infinite M200 instrument (Männedorf, Switzerland) was used for recording the fluorescence spectrum and for the fluorescence measurement of the substrates.

*RAMAN*: UV Resonant Raman (UVRR) measurements have been carried out at the Elettra synchrotron radiation facility. A complete description of the experimental apparatus can be found elsewhere [36]. A 266 nm laser source has been employed. The beam reaching the sample was approximately 25 μW. The Raman scattering signal was collected with a backscattering configuration. A Czery-Turner spectrometer with focal length of 750 mm, coupled with a holographic reflection grating of 1800 g/mm and with a Peltier-cooled back-thinned CCD was employed to get the Raman signal. Spectral resolution was set to 8 cm^−1^. Raman frequencies were calibrated by means of cyclohexane spectra [37]. The samples have been functionalized with commercial salmon sperm DNA (Sigma-Aldrich). It was diluted in pure water up to a concentration of 10 μg/mL (i.e., 33 μM on nucleobases molar concentration), then deposited by drop casting (drop of 5 μL) on a rough Aluminum and on an NPA sample. Considering 1 mm^2^ of surface covered by the drop, it corresponds to a superficial concentration of 20 nucleobases per nm^2^ in case of homogenous distribution when water is evaporated.

*FEM simulations*: To explore the plasmonic properties of porous Al in the UV range, a numerical investigation of the electromagnetic response of such material has been conducted. A finite element method (FEM) commercial software, COMSOL Multiphysics, has been employed to model the optical behavior of the nanoporous metal. In particular, following a procedure we recently reported [5], nanometric pores and irregularities of NPA have been numerically reproduced by means of SEM images from experimental samples. To resolve nanometric features of the numerical solution for the electromagnetic fields, domains are finely meshed: Within the porous domains, mesh elements have a maximum allowed size of 10 nm and a minimum size equal to 2.5 nm. In the surrounding environment, the maximum element size is equal to λ/5, which ensures proper resolution of propagating fields in homogeneous media. Free triangular elements have been defined to mesh the two-dimensions domains. Due to their crucial role in generating the highly localized field enhancement, a proper description of the material’s features is strictly necessary. Accordingly, the brightness of SEM images of the metal surface cross-sections is used to encode the information concerning the profile of a nanoporous film. Upon uploading in the software environment, a SEM image of the metal surface is treated as a two-dimensional function, with values between 0 (where there is no metal) and 1 (where pure metal optical properties are considered), which defines a map of permittivity of the modelled geometry. NPA can be, thus, optically described by the following 2D weighted permittivity function:

$$\varepsilon \;=\;\{\begin{array}{c} \varepsilon_{metal}\;*\;map\left(x,\;\;y\right)\;\mathrm{if}\;map\left(x,\;\;y\right)\;>\;ths\\ \varepsilon_{background}\;\mathrm{if}\;map\left(x,\;\;y\right)\;<\;ths \end{array}$$

where map(*x*, *y*) is the spatial function built from the SEM image of the NPA cross section, ε_background_ the dielectric constant of the considered surrounding environment (here air, therefore equal to 1), *ε_metal_* the dielectric function of the metal (Al [38] for NPA, Au [39] for nanoporous gold (NPG) used a standard for comparison) and *ths* a threshold value, used to define the numerical boundary between metal and background in the geometry, and chosen to maximize the resemblance between model and sample. In the present work, the value *ths* = 0.6 has been set to achieve the most realistic profile. The electromagnetic problem is solved in frequency domain for λ = 260 nm and λ = 350 nm. A port analysis has been employed to simulate an impinging monochromatic planewave, propagating in the direction perpendicular to the surface and polarized along the direction parallel to the NPA substrate. Continuity periodic boundary conditions and perfectly matched layers have been defined on the lateral sides and on both the top and bottom of the structure, respectively. Finally, to be noted, the presence of the oxide is currently accounted for implicitly in the choice of *ths* in order to replicate the geometrical features observable in the SEM images.

## 3. Results and Discussion

As previously mentioned, Yang et al. [32] have successfully converted foils of Al_2_Mg_3_ into NPA by exposing them to an ionic liquid. The GRR proceeds as follows:

Al_2_Mg_3_ + 2 AlCl_4_^−^ > 4 Al(s) + 3 Mg^2+^ + 8 Cl^−^

This system shows very peculiar properties from the standpoint of materials preparation: (i) The GRR causes exhaustive replacement of Mg by Al; (ii) oxide formation and hydrogen evolution are excluded or minimized by operation in ionic liquid; (iii) Al deposition occurs inward, towards the bulk of the foil loosing Mg, and not outwards (towards the electrolyte) as one would normally expect; (iv) the process is a homogeneous plating with fresh Al of the inner nanostructures resulting from Mg loss and Al atoms rearrangement. The resulting product is a bulk, porous, rather pure Al layer. A crucial role for the inward-growth appears to result from two facts: (i) The volume of solid phase decreases during GRR, leaving space for electrolyte permeation of the porous structure and mass transport in the pores; (ii) the metal ion is carried by negatively charged (anionic) species, moving inwards towards the receding reaction front.

### 3.1. Structural Properties

**Figure 1:**
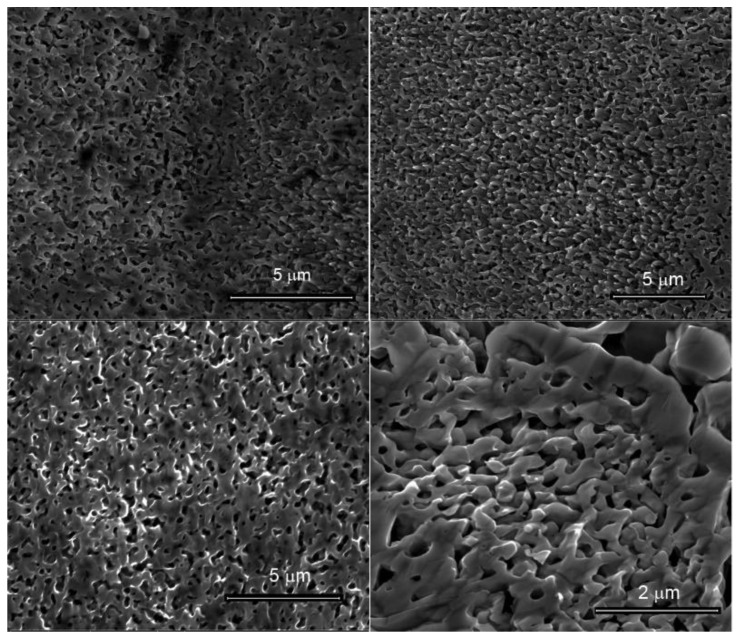
SEM micrographs of the galvanic replacement reaction (GRR) sample, displaying different area of the same sample.

Figure 1 shows some examples of NPA morphology obtained from GRR by employing the alloy prepared from melted Al and Mg. The obtained morphology confirms previous results reported in [32]. Comparing this sample with porous structures obtained by means of chemical dealloying [7], nanoporous Al/Mg prepared via dealloying from a co-sputtered film shows pores of dimension down to a few tens of nm, which can be modulated acting on the starting composition of the alloy and on the dealloying condition. The samples obtained by means of GRR, on the contrary, are less uniform in terms of morphology and present pores that are an order of magnitude larger. In order to verify that the different morphology was not due to the different procedure of deposition of the alloy (in the previous case we used co-sputtering deposition from Al and Mg targets), chemical dealloying in acetic acid has been here performed on Al_2_Mg_3_ prepared via melting. The results, reported in Supporting Information (Note #1) confirm that, while it’s possible to achieve nanoporous films with pores down to tens of nm, the chemical dealloying process does not allow the complete etching of Mg, and the final oxide contents within the porous samples are always significant.

The compositions, obtained from EDS, are reported in Table 1. The oxygen content around 14% are only related to the ambient exposure of the prepared film and its stable in time. This is a first proof that the obtained material is metallic Al. More importantly, the GRR enables the complete etching of Mg from the starting alloy, as confirmed from the XRD analysis reported in SI (Figure S2). The complete removal of Mg has not been possible with the chemical dealloying [7], and this result demonstrates that the GRR actually get a nanoporous Al structure.

The strong air reactivity of both Al and Mg suggests the oxidation of the obtained sample surfaces. The analysis of the film surface (within the first 10 nm) oxygen content can be obtained by means of XPS, as reported in Table 2 (details of the measured spectrum are reported in SI Note#2).

### 3.2. Optical and Plasmonic Properties

**Figure 2:**
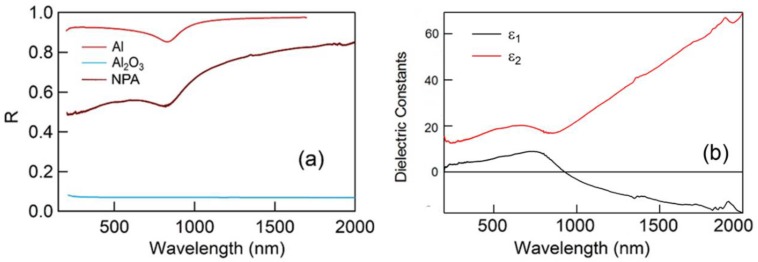
(**a**) Reflectance spectra of Al, Al_2_O_3_, and nanoporous Al (NPA), respectively; (**b**) Dielectric Constants of NPA obtained from Kramers-Kronig relationship.

The first characterization of the NPA optical performances has been done by measuring the reflectance in the spectral range between 200 and 2000 nm. Due to the high roughness of the samples, in order to collect the reflectance spectrum, a spectrophotometer equipped with an integrating sphere has been used. In this way, the direct and diffuse reflectance can be collected. Figure 2 reports the spectrum obtained from the described samples in comparison with the experimental spectrum from Al and Al_2_O_3_ reference samples. This allows a clear evaluation of the features related to the high porosity and to the oxidation of the sample. The presence of an absorption band at 800 nm, ascribable to the Al interband transition, also confirms the pure metallic structure of the film. In principle, the reflectance spectrum can be used to obtain an estimation of the dielectric constants of the samples [40]. Anyway, the estimation of the dielectric function of our NPA sample is not trivial. In fact, the exploitation of the effective medium theory is prevented because the assumptions of the ligaments size are much smaller than the wavelength is not fulfilled. This is justified by the need of the integrating sphere to take into account all the signals diffused by the rough NPA surface. For the same reason, approximating the electrodynamical behavior of NPA electron by the Drude–Lorentz model and calculating the reflection coefficient of the NPA/Air interface by the Fresnel equations is unreliable because the latter assumes the interface between the media is flat and that the media are homogeneous and isotropic. Given all the above considerations, the Kramers-Kronig [41] relations have been used as the most reliable procedure to retrieve the dielectric function from the measured spectra. While the details on the application of the Kramers-Kronig relationship to our data are reported in SI (Note#3), the result of this approach is shown in Figure 2B, where the NPA dielectric function is plotted as a function of the wavelength.

This data describes the average response of the material, but the porous structure and the consequent localized plasmonic effect can be described only considering the actual morphology of the material (see section below).

As previously stated [7], these nanoporous samples prepared in metallic Al can find applications in field-enhanced spectroscopies. In particular, enhanced fluorescence (FE) and SERS can be probed in order to evaluate the potential performances of the prepared films.

**Figure 3:**
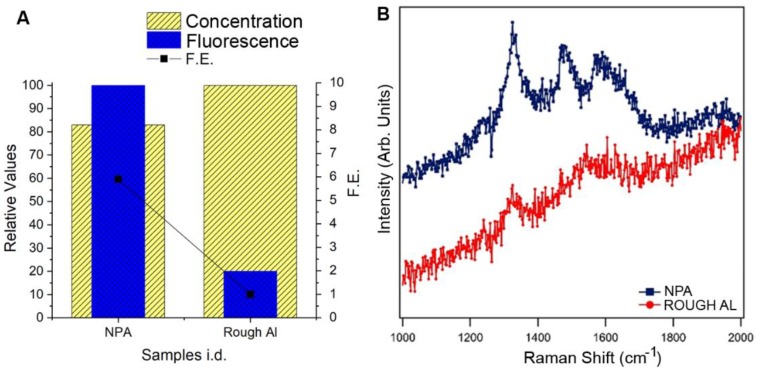
(**A**) Experimental FE in GRR NPA compared to standard rough Al sample. (Yellow bars report the measured concentration of dyes linked on the surface; blue bars report the measured fluorescence emitted from the functionalized samples); (**B**) experimental UVRR spectra of salmon sperm DNA deposited by drop casting on rough Al (red curve) and NPA (blue curve) substrates.

In order to perform FE experiments, the NPA samples have been functionalized according to the protocol reported in Methods. In particular, we chose to use Coumarins as reported dyes. They are well-known dyes, widely exploited for their optical properties and for the development of “off-on” switchable fluorescent biosensors [42]. 7-hydroxy-4-coumarin acetic acid was selected for the evaluation of the FE, achieved with the discussed alumina-based porous substrates due to its fluorescent properties. Firstly, this dye absorbs in the UV range (360 nm), and secondly, it has a suitable Stokes shift (100 nm) which makes it easier to measure the fluorescence of the dye on the substrate, hence avoiding interferences related to the substrate itself. Furthermore, this dye already proved its applicability for the development of switchable sensors, therefore, the possibility of enhancing its fluorescence signal could potentially lead to intriguing improvements in terms of sensitivity and limit of detection (LOD) of such fluorescent-based detection approaches [43]. The substrate was firstly treated with APTES (3-Aminopropyl) triethoxysilane) in order to have amino groups exposed on the surface. These amino groups were subsequently used as anchors to covalently attach the carboxylated-coumarin previously activated using EDC-NHS as coupling agents. A scheme of the different functionalization steps can be found in [7]. The same protocol was performed on NPA samples and on substrate made of rough Al [28,29], used as reference. This allows us to compare the FE achievable with NPA and a well-known film with good properties in the UV spectral range. The comparison was accomplished considering both the fluorescent signal measured and the corresponding amount of dye effectively attached on the surface. In particular, we evaluated the enhancing efficiency by means of the FE factor calculated by dividing the fluorescent signal by the calculated amount of coumarin present at the surface. The FE factor is normalized to the value determined for rough Al (see SI Note#4). The FE factor shown in Figure 3A defines the relationship between concentration of dye and fluorescent signal, allowing to determine the fluorescence enhancement for each substrate here considered. As noticeable in Figure 3A, the FE was about 6 folds with respect to the reference sample.

The potential application of the prepared NPA substrate as SERS platform has been evaluated by measuring the UV Raman spectrum from commercial DNA. The concentration used for the functionalization of NPA and rough Al reference samples correspond to about 20 nucleotides per nm^2^. Figure 3B shows the UVRR spectrum, obtained by salmon sperm DNA deposited on reference Al and on NPA. Each spectrum corresponds to an average of 8 spectra collected in different positions of the drop, equally distributed from the center to the border of the drop limit. Both spectra show the typical DNA vibrational features. More specifically the peaks at 1337 cm^−1^ are addressed to adenine, the ones at 1484 cm^−1^ and 1580 cm^−1^ to both adenine and guanine, while the bump at 1655 cm^−1^ is mainly a thymine contribution [44,45]. The NPA peak results enhanced by a factor at least of 5 with respect to those of the spectrum acquired on reference Al surface.

To be noted, the obtained enhancement in FE and in SERS are even more significant if we consider that the rough Al samples used as reference are known to enhance both the fluorescence [28] and the Raman signal [29] in the UV spectral region.

### 3.3. Numerical Simulations

**Figure 4:**
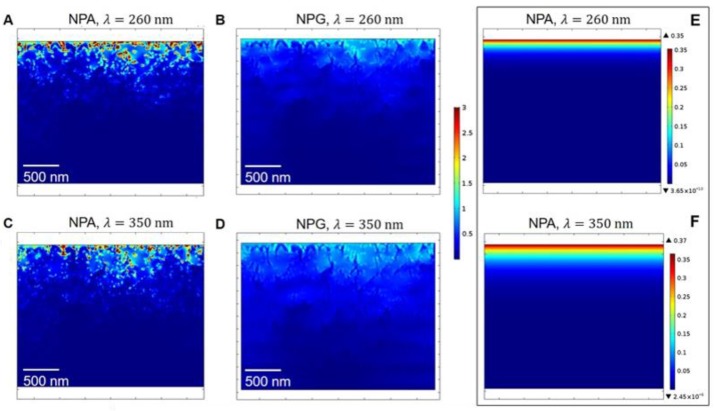
Numerically-computed field enhancement in NPA (**A**,**C**) and nanoporous gold (NPG) (**B**,**D**) films, at excitation wavelengths λ = 260 nm (**A**,**B**) and λ = 350 nm (**C**,**D**). Electric fields are calculated following the procedure introduced in the Methods section. Simulated electric fields at 260 nm (**E**) and 350 nm (**F**), according to the homogenous dielectric constant obtained from experimental measurements, as in Figure 2.

The optical behavior of NPA in the UV range has been investigated numerically. The field confinement induced by the nanoporous structure of the film is determined by means of a 2D electromagnetic computation (see Methods for details), and its spatial distribution is shown in Figure 4 across a sample section, for different values of excitation wavelength. The same computation has been performed on nanoporous gold (NPG). Results highlight the better performances achieved with NPA in the considered spectral range, when compared to standard plasmonic materials, such as Au. Following procedure similar to the one we recently reported [5], the quantity $\left|E/E_{0}\right|$ is computed across the same geometry (plots with the comparison between |E| and |E|^2^ maps are reported in SI Note#5, Figure S6), generated by using the same SEM image of the experimental sample, but optically described by Al [38] (Figure 4A,C) and Au [39] (Figure 4B,D) dielectric function, respectively (important to note, no oxidation effects have been explicitly considered here). To further stress the higher localization of the field in the case of NPA, scales for field enhancement of Figure 4 are also the same. This result clearly proves the improvement achieved in using NPA instead of NPG. Indeed, for a given nanostructure of pores and spatial features of a surface, a much higher confinement can be induced in the considered spectral region when Al is used.

Hence, these simulations have been useful to confirm what was experimentally observed in terms of the NPA optical properties. Moreover, the comparison between Figure 4A,B,E,F should highlight the importance of the modelling approach for nanoporous materials. Figure 4A,B have been obtained by following the approach detailed in the Methods section (namely, the NPA is described by a weighted permittivity map based on (i) the experimental sample SEM image (ii) standard Al permittivity [38]). Figure 4E,F, instead, show the results for field enhancement, where the NPA is homogeneous and optically described by the average permittivity function measured for nanoporous Al (i.e., not standard Al). From a macroscale perspective, the latter approach should be able to account for the porous nature of NPA, as the former. However, our modelling approach can better support experimental evidences, which crucially depend on the nanometric features of the surface. To this extent, our simulations should be useful. Indeed, the comparison between Figure 4A,B and Figure 4E,F shows a more relevant penetration of the incident field across the structure in the former case than in the latter. This is directly linked to the porosity of the surface. In the case of Figure 4E,F, the NPA essentially behaves as a flat metal where, although the utilized dielectric function accounts for the structure porosity, penetration is ruled only by the metal skin depth, δ = λ/4 πκ, with κ the imaginary part of the refractive index, of the order of few tens of nm (additional simulations are reported in SI Note#5). However, in Figure 4A,B, nanopores play a role in the interaction of the field with the surface, and the skin depth cannot be strictly defined as in the former case, due to the presence of the surrounding environment (here air) at the interface. From the standpoint of simulations, the presence (and therefore its effect on the optical behavior of the system) of air in the Al pores is governed and controlled by the parameter *ths*, which states the nanoporous surface boundary.

Finally, it’s worth mentioning that the excitation of LSPRs in metallic nanostructures have the inherent problem of heating. This is an important problem in SERS, since the sample can be vaporized. A rigorous approach for heat transport in nanoporous materials requires a deeper knowledge of NPA thermal properties. However, an approximation of the induced temperature increase can be done taking into consideration the used beam size and laser power. Details of this analysis can be found in SI Note#5.

## 4. Conclusions

In conclusion, here we reported on GRR based procedures to prepare NPA films from an alloy of Al and Mg. Although present, the oxide layer is stable in time and under it a porous structure of metallic Al is always present. Optical spectroscopies have been used to evaluate the dielectric constants of the prepared NPA and to verify its potential applications as UV plasmonic material. In particular, we verified that NPA films can be used as an interesting platform, both in UV fluorescence and UV SERS. Enhancement factors about 5 with respect to a standard rough Al sample have been observed in both cases.

## Figures and Tables

**Table 1:** Initial composition x, composition after the GRR as measured by means of energy-dispersive spectroscopy (EDS).

Sample	Pristine Composition × (Mg*_x_*Al_1−*x*_)	(EDS) Etched Composition (O, Al, Mg)
NPA	0.6	14%, 84%, 0%

**Table 2:** XPS analyses.

Sample	Al_2_O_3_ (at%)	Al Suboxides (at%)	Metallic Al	MgO
NPA	68.9%	10.5%	20.6%	--

## Supplementary Materials

The following are available online at https://www.mdpi.com/2079-4991/10/1/102/s1, Note#1: Chemical Dealloying of melted Al_2_Mg_3_; Figure S1. SEM micrographs of NPA samples prepared from the melted alloy via chemical dealloying in acetic acid; (A) dealloying for 30 min; (B) dealloying for 3h and 30min; (C) dealloying for 7h. Table S1. Samples, initial composition x, composition after the GRR as measured by means of EDS. Figure S3. XPS analysis for the NPA sample. Note#2 XRD and XPS spectrum of NPA prepared via GRR; Figure S2. XRD analysis for the NPA sample. Note#3 Dielectric Constants from Kramers-Kronig relationship; Note#4 Fluorescence measurements data; Table S2. Experimental data from fluorescence measurements. Note#5 Additional numerical simulations. Figure S4. Comparison in penetration depth as predicted with two different calculation approaches. The norm of the electric field is plotted along a vertical line through the NPA region cross section (red arrow). The case of nanoporous and flat geometry are compared at two wavelengths, 260 nm and 350 nm. In both cases, the nanoporous geometry predicts larger penetrations of the electric field in the NPA. Here the penetration ($\delta_{\mathrm{nano}}$ and $\delta_{\mathrm{flat}}$ for the two numerical approaches) is assumed, for simplicity, the distance from the surface at which the field is decreased to 10% of its incident value. Figure S5. 2D map of the electric field in the NPA section (left). 2D map of the dissipation in the NPA section in log scale (middle). Temperature map of the full sample: air+NPA layer+Al substrate. Incident beam size is 500 nm and input power is 25 μW. Figure S6. comparison of |E| and |E|^2^ maps for NPA at λ=260 nm. Both large and closed up views are presented. Colors are saturated to better visualize the features of the field. Maximum values in each case are also reported in the scale bars for both cases.
