# Supplementary material for: Galvanic Replacement Reaction as a Route to Prepare Nanoporous Aluminum for UV Plasmonics
Source: Nanomaterials (Basel). 2020 Jan 4;10(1):102. doi: 10.3390/nano10010102 (PMC7023067; doi:10.3390/nano10010102)
Supplement: Supplementary file 1 [file nanomaterials-10-00102-s001.pdf]

Supporting Information

Galvanic Replacement Reaction As A Route To Prepare Nanoporous Aluminum for UV Plasmonics

Denis Garoli [1,*], Andrea Schirato [1,2], Giorgia Giovannini [3], Sandro Cattarin [4], Paolo Ponzellini [1], Eugenio Calandrini [1], Remo Proietti Zaccaria [1,5], Francesco D'Amico [6], Maria Pachetti [6,7], Wei Yang [8], Hai-Jun Jin [8], Roman Krahne [1] and Alessandro Alabastri [9]

[1] Istituto Italiano di Tecnologia, via Morego 30, I-16163 Genova, Italy; andrea.schirato@polimi.it (A.S.); paolo.ponzellini@iit.it (P.P.); eugenio.calandrini@iit.it (E.C.); remo.proietti@iit.it (R.P.Z.); roman.krahne@iit.it (R.K.)
[2] Deparment of Physics, Politecnico di Milano, Piazza L. da Vinci 32, I-20133 Milan, Italy
[3] EMPA Federal Swiss research Institute, 9014 St. Gallen, Switzerland; giorgia.giovannini@empa.ch
[4] ICMATE—CNR, Corso Stati Uniti 4, 35127 Padova, Italy; sandro.cattarin@cnr.it
[5] Cixi Institute of Biomedical Engineering, Ningbo Institute of Industrial Technology, Chinese Academy of Sciences, 1219 Zhongguan West Road, Ningbo 315201, China
[6] Elettra Sincrotrone Trieste S.C.p.A., S.S. 14 km 163,5 in Area Science Park, 34149 Basovizza TS, Italy; francesco.damico@elettra.eu (F.D.A.); maria.pachetti@elettra.eu (M.P.)
[7] Department of Physics, University of Trieste, Via Alfonso Valerio 2, 34127 Trieste, Italy
[8] Shenyang National Laboraory for Materials Science, Institute of Metal Research, Chinese Academy of Sciences, 72 Wenhua Road, Shenyang 110016, China; wyang15s@imr.ac.cn (W.Y.); hjjin@imr.ac.cn (H.-J.J.)
[9] Department of Electrical and Computer Engineering, Rice University, 6100 Main Street MS-378, Houston, TX 77005, USA; alessandro.alabastri@rice.edu
* Correspondence: denis.garoli@iit.it

Received: 4 December 2019; Accepted: 31 December 2019; Published: date

Supporting Note #1 – Chemical Dealloying of melted Al_2 Mg_3

In order to compare the results obtained from GRR, chemical dealloying in acetic acid 1M (in Methanol) has been performed on the same starting alloy. Different durations of the dealloying have been tested: 30 minutes, 3 hours and 7 hours. The results, in terms of morphology and composition are reported in Fig. S1 and in Table S1. As can be observed the complete removal of Mg is not possible and the oxide level is high (the high level of oxidation makes also hard to collect a high resolution SEM micrograph in the case of the longest dealloying – Fig. S1C).

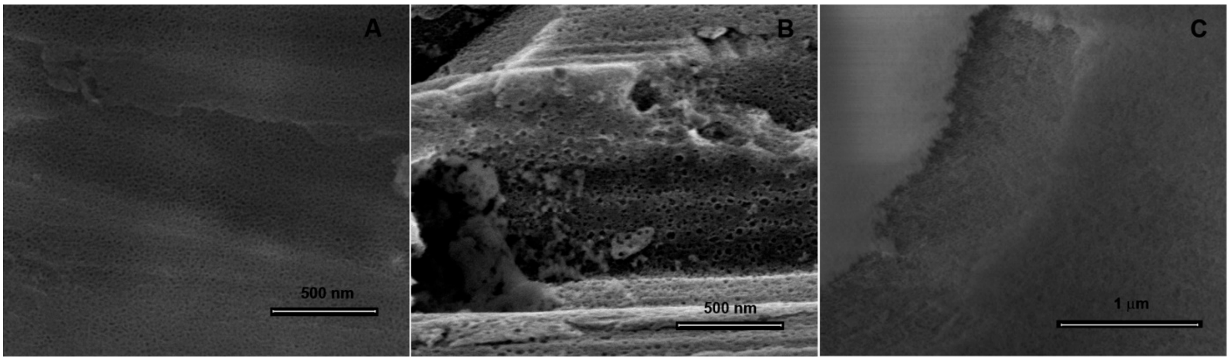

Figure 1. SEM micrographs of NPA samples prepared from the melted alloy via chemical dealloying in acetic acid; (A) dealloying for 30 min; (B) dealloying for 3h and 30min; (C) dealloying for 7h.

Table S1. Samples, initial composition x, composition after the GRR as measured by means of EDS.

Samples	Dealloying duration	(EDS) Etched composition (O, Al, Mg)
D_NPA1	30 min	45%, 28%, 27%
D_NPA2	3h 30 min	68%, 28%, 4%
D_NPA3	7h	72%, 26%, 2%

Supporting Note #2 – XRD and XPS spectrum of NPA prepared via GRR

XRD measurements show four peaks all associated to Al:

Al [111] 2θ:38,5 ° ; Al [002] 2θ: 44,7 ° ; Al [022] 2θ: 65,1 ° ; Al [113] 2θ: 78,2 °

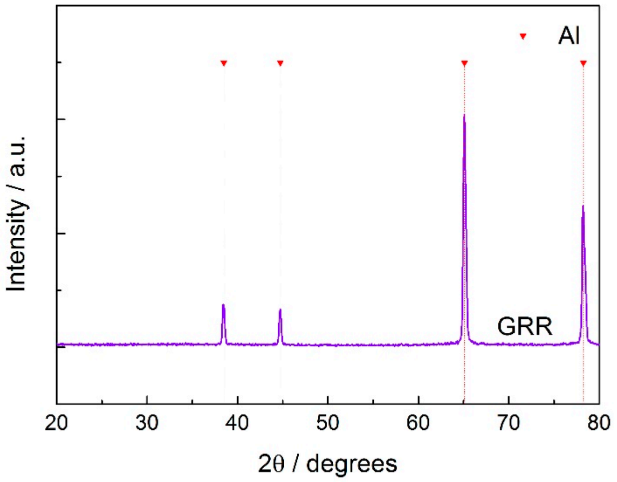

Figure S2. XRD analysis for the NPA sample.

The XPS analysis (Fig. S3) is affected by the superficial oxidation of the sample, that occurs due to its exposure to the air. After a first XPS measurement, the sample was sputtered inside the XPS machine chamber, to unveil the not-oxidized material under the surface, but apparently the sputtering time was insufficient, yielding an oxidation value that is just slightly lower than the one measured for the as prepared GRR sample.

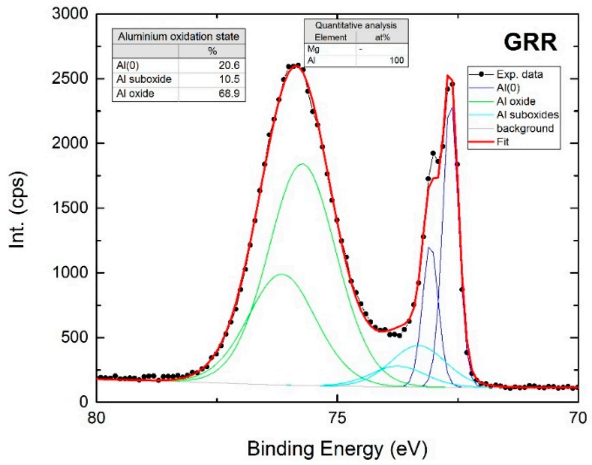

Figure S3. XPS analysis for the NPA sample.

Supporting Note #3 – Dielectric Constants from Kramers-Kronig relationship

The Kramers-Kronig (KK) relations can be used to derive various expressions connecting the real and imaginary parts of different optical parameters. In the present case, since the only experimental observable was the reflectivity of the NPA films, we employed the KK relations to derive the phase shift $\theta(\omega)$ of an electromagnetic wave due to the reflection on the NPA surface. In formulas

$$\theta(\omega) = -\frac{2\omega}{\pi}\int_0^{\infty}\frac{\ln\sqrt{R(\omega')} - \ln\sqrt{R(\omega)}}{\omega'^2 - \omega^2}d\omega'$$

Recalling that the measured reflectivity is defined by the square modulus of the complex reflection coefficient

$$R(\omega) = |\tilde{r}|^2$$

And the Fresnel equations

$$\tilde{r} = |\tilde{r}|e^{i\theta} = \frac{1-\tilde{n}}{1+\tilde{n}}$$

We can find an expression for $\tilde{n}$

$$\tilde{n} = \frac{1 - R - 2Im(\tilde{r})}{1 + R + 2Re(\tilde{r})}$$

Finally, to derive the dielectric function we use $\tilde{n} = \sqrt{\tilde{\varepsilon}}$.

Since the integral in the first equation is performed over the entire frequency range and we are able to measure the reflectivity in a defined frequency window, we have to extrapolate the data in the uncovered range. For the low energy extrapolation, the spectra are extrapolated by the Hagen-Rubens [1], as expected for metallic samples. For the high energy extrapolation, the reflectance spectra are extrapolated as constant up to 25eV (200000 cm^{-1}) and above this energy according to the behaviour of free electrons (about ω^{-4}). It is important to notice from the integral that regions such as $\omega' \ll \omega$ or $\omega' \gg \omega$ has minor contributions to $\theta(\omega)$.

Supporting Note #4 – Fluorescence measurements data

The following table reports the experimental values obtained from fluorescence measurements.

Table S2. Experimental data from fluorescence measurements.

	μM	Fluorescence	E	E normalized	μM relative	Fluorescence relative
**Rough Al**	382,88	2820,00	7,365	1,000	100	20
**NPA**	316,96	13943,00	43,990	5,973	83	100

The enhancement (E) is calculated with respect to the same measurements performed on a Silicon substrate where no enhancement is expected. The concentration is calculated as described in Method section.

Supporting Note #5 – Additional numerical simulations

Penetration of the norm of the electric field along the vertical direction orthogonal to the surface

We report here, for comparison, the penetration of the norm of the electric field along the vertical direction orthogonal to the surface, in the case of nanoporous (as in Figs.4 A,C) and homogeneous Al geometry (as in Figs.4 E,F) (δ_{nano} and δ_{flat}). Due to the presence of air domains, the calculations based on the realistic nanoporous geometry predict an overall larger penetration of the electric field in the NPA at both tested wavelengths (260 nm and 350 nm).

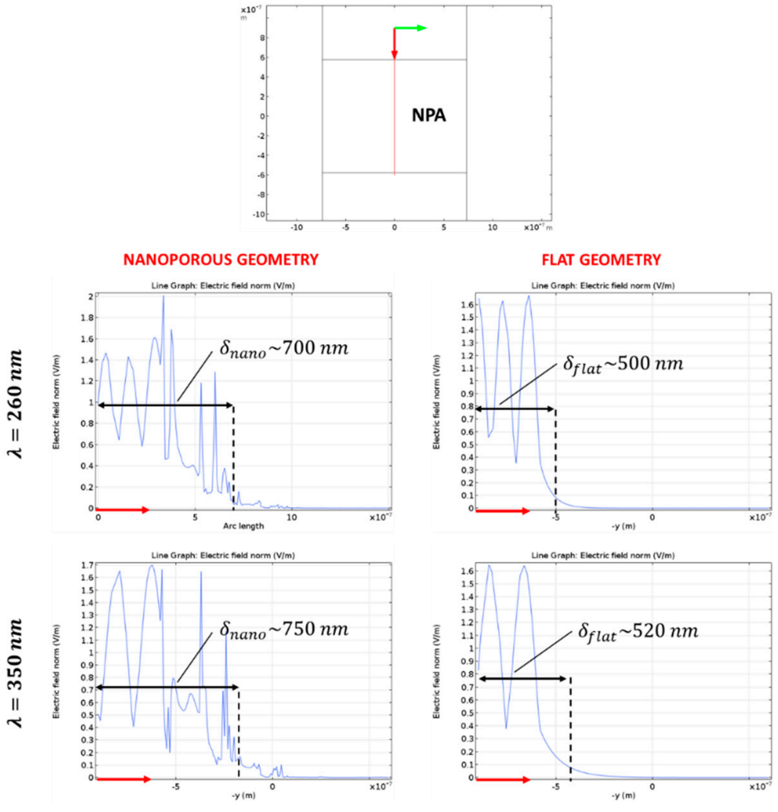

Figure 4. Comparison in penetration depth as predicted with two different calculation approaches. The norm of the electric field is plotted along a vertical line through the NPA region cross section (red arrow). The case of nanoporous and flat geometry are compared at two wavelengths, 260 nm and 350 nm. In both cases, the nanoporous geometry predicts larger penetrations of the electric field in the NPA. Here the penetration (δ_{nano} and δ_{flat} for the two numerical approaches) is assumed, for simplicity, the distance from the surface at which the field is decreased to 10% of its incident value.

Thermal response of the NPA substrate

To calculate the approximated thermal response of the NPA substrate, we have mapped the 2D absorption, Q, at $\lambda = 260$ nm calculated following Joule dissipation, $Q = \mathbf{J} \cdot \mathbf{E}$, on a 2D axisymmetric geometry which allows us to introduce the realistic circular shape of the impinging beam. The beam size (radius) utilized during the experiment is $w_0 = 0.5\ \mu m$ and the input power is $P_{in} = 25\ \mu W$. We have utilized the available SEM images to reproduce the 3D heat propagation in a 2D axisymmetric geometry. While this approach fully reproduces the porous features only within vertical sections, we believe it represents a fair starting point for thermal evaluations. A full 3D calculation would require many different SEM tomographic sections. In Fig. S5, we report the electric field distribution, heat dissipation and temperature profile of the calculate NPA structure. A final temperature of ~315 K is calculated. The irregularities in the temperature maps are due to the different values of the air ($k_{air} = 0.025\ W/(m \cdot K)$) and aluminium ($k_{Al} = 238\ W/(m \cdot K)$) thermal conductivities which alternate within the structure, according to the same mapping procedure utilized for the structure's optical properties (see equation for ε in the main text).

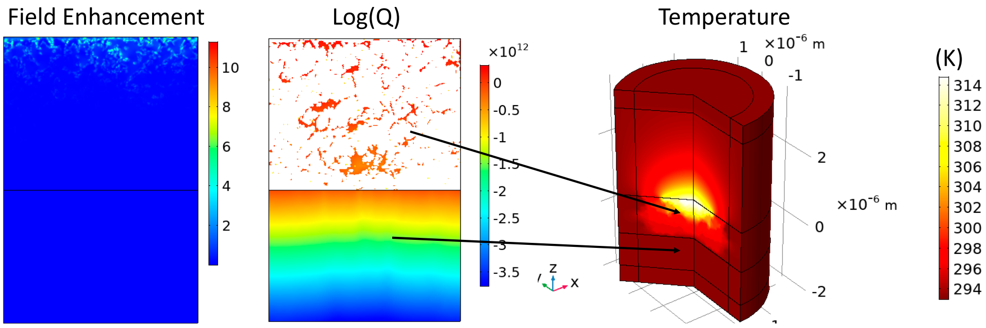

Figure 5. 2D map of the electric field in the NPA section (left). 2D map of the dissipation in the NPA section in log scale (middle). Temperature map of the full sample: air+NPA layer+Al substrate. Incident beam size is 500 nm and input power is 25 µW.

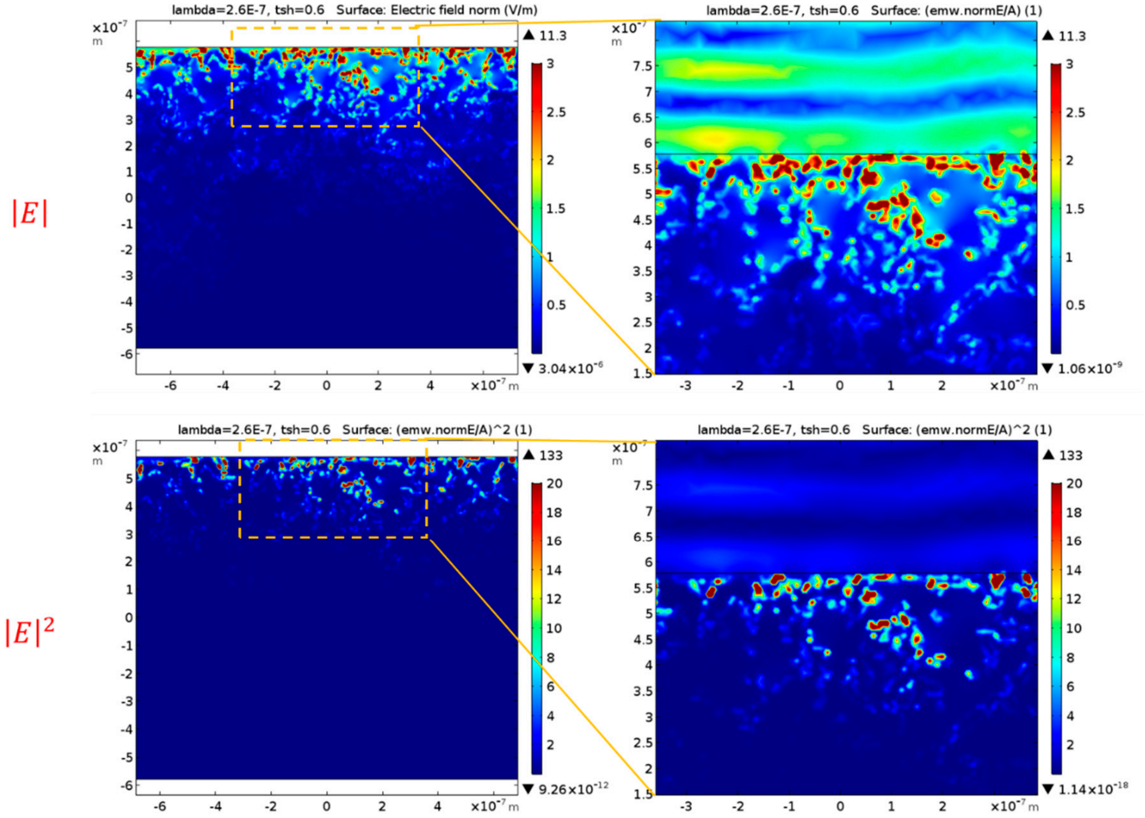

Figure 6. comparison of |E| and |E|² maps for NPA at λ = 260 nm. Both large and closed up views are presented. Colors are saturated to better visualize the features of the field. Maximum values in each case are also reported in the scale bars for both cases.

References

1. Dressel, M.; Grüner, G. *Electrodynamics of solids: optical properties of electrons in matter*; Cambridge University Press, 2002; ISBN 0521592534.
